# Imipenem/Relebactam Ex Vivo Clearance during Continuous Renal Replacement Therapy

**DOI:** 10.3390/antibiotics10101184

**Published:** 2021-09-29

**Authors:** Soo Min Jang, Lenar Yessayan, Michael Dean, Gabrielle Costello, Ravi Katwaru, Bruce A. Mueller

**Affiliations:** 1 Department of Pharmacy Practice, Loma Linda University School of Pharmacy, Loma Linda, CA 92350, USA; 2 Department of Medicine Division of Nephrology, Michigan Medicine, Ann Arbor, MI 48109, USA; lenar@med.umich.edu; 3 Chicago College of Osteopathic Medicine, Midwestern University, Downers Grove, IL 60515, USA; Michael.dean@midwestern.edu; 4 College of Human Medicine, Michigan State University, East Lansing, MI 49503, USA; coste103@msu.edu; 5 Merck & Co., Kenilworth, NJ 07033, USA; ravi.katwaru@merck.com; 6 Department of Clinical Pharmacy, University of Michigan College of Pharmacy, Ann Arbor, MI 48109, USA; muellerb@med.umich.edu

**Keywords:** CRRT, imipenem, relebactam, pharmacokinetics, clearance

## Abstract

(1) Purpose of this study: determination of adsorption and transmembrane clearances (CL_TM_) of imipenem and relebactam in ex vivo continuous hemofiltration (CH) and continuous hemodialysis (CHD) models. These clearances were incorporated into a Monte Carlo Simulation (MCS), to develop drug dosing recommendations for critically ill patients requiring continuous renal replacement therapy (CRRT); (2) Methods: A validated ex vivo bovine blood CH and CHD model using two hemodiafilters. Imipenem/relebactam and urea CL_TM_ at different ultrafiltrate/dialysate flow rates were evaluated in both CH and CHD. MCS was performed to determine dose recommendations for patients receiving CRRT; (3) Results: Neither imipenem nor relebactam adsorbed to the CRRT apparatus. The CL_TM_ of imipenem, relebactam, and urea approximated the effluent rates (ultrafiltrate/dialysate flow rates). The types of hemodiafilter and effluent rates did not influence CL_TM_ except in a dialysis flow rate of 1 L/h and 6 L/h in the CHD with relebactam (*p* < 0.05). Imipenem and relebactam 200 mg/100 mg every 6 h were sufficient to meet the standard time above the MIC pharmacodynamic targets in the modeled CRRT regimen of 25 kg/mL/h. (4) Conclusions: Imipenem and relebactam are not removed by adsorption to the CRRT apparatus, but readily cross the hemodiafilter membrane in CH and CHD. Dosage adjustment of imipenem/relebactam is likely required for critically ill patients receiving CRRT.

## 1. Introduction

Imipenem/relebactam, a novel combination of carbapenem/dehydropeptidase and beta-lactamase inhibitor, has demonstrated activity against multi-drug resistant (MDR) strains of *P. aeruginosa* and many *Klebsiella pneumoniae* carbapenemases (KPC)-producing *Enterobacteriaceae* in previous in vitro studies [1,2,3]. The emergence of MDR pathogens such as *P. aeruginosa, Escherichia coli, Klebsiella pneumoniae, and Enterobacter spp.* limit the use of many antibiotic agents [4], because antibiotics are generally not effective against these pathogens [5]. The pharmacokinetics of imipenem/cilastatin in critically ill patients receiving CRRT have been previously studied [6,7,8,9]. However, there are no published imipenem/relebactam pharmacokinetic data from critically ill patients receiving CRRT. Moreover, clearance by adsorption must also be considered, since drugs, such as colistin, can adsorb to the CRRT apparatus [10,11]. The purpose of this study was to determine adsorption and transmembrane clearances (CL_TM_) of imipenem and relebactam in ex vivo continuous hemofiltration (CH) and continuous hemodialysis (CHD) models. Cilastatin disposition was not assessed in this study.

## 2. Results

**Figure 1:**
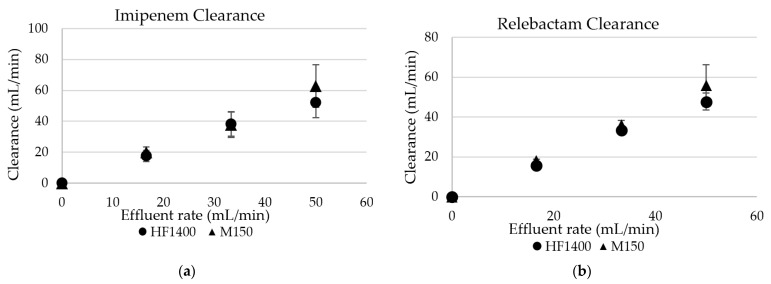
(**a**) Imipenem continuous hemofiltration transmembrane clearance. (**b**) Relebactam continuous hemofiltration transmembrane clearance.

**Figure 2:**
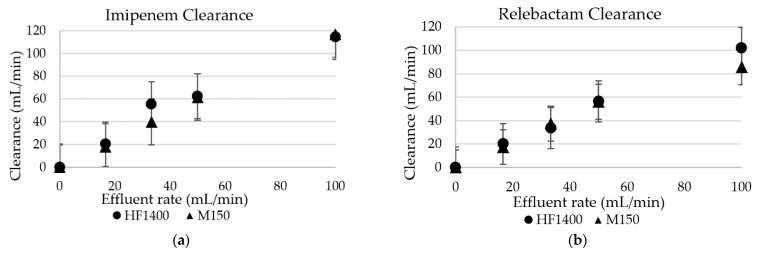
(**a**) Imipenem continuous hemodialysis transmembrane clearance; (**b**) Relebactam continuous hemodialysis transmembrane clearance.

No imipenem/relebactam degradation was observed in the 37 °C blood after 1 h. Neither imipenem nor relebactam adsorption was observed with either hemodiafilter type. The CL_TM_ of urea, imipenem, and relebactam approximated the effluent rates [the saturation coefficient (SA) and sieving coefficient (SC) consistently approximated 1 for all three solutes]. Sieving coefficients and saturation coefficients are summarized in Table 1. During CH, the CL_TM_ were dependent on the ultrafiltration rate (Quf) (Figure 1a,b). The change in Quf did not influence the sieving coefficient. The hemodiafilter type did not influence the CL_TM_ (*p*-value > 0.05), either. The CL_TM_ were dependent on the dialysate flow rate during CHD (Figure 2a,b). During CHD, the hemodiafilter type influenced the CL_TM_ for relebactam (*p*-value < 0.05) at dialysate rates (Qd) of 17 mL/min and 100 mL/min. This ex vivo study indicates that imipenem/relebactam is readily cleared by CH and CHD (Figure 2 and Table 2).

**Figure 3:**
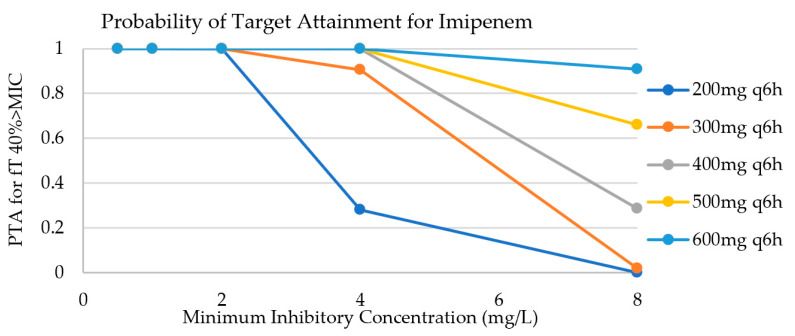
Probability of target attainment for imipenem dosing regimens, for a CRRT effluent rate of 25 mL/kg/h. Relebactam was not included in the figure, because it reaches a PTA ≥ 90% at all times.

The Monte Carlo simulation (MCS) results for three pharmacodynamic (PD) targets 40% free concentration time above minimum inhibitory concentration (fT > 1 × MIC), 40% fT > 4 × MIC and 100% fT > 1 × MIC of imipenem are summarized in Table 2. Figure 3 illustrates the probability of target attainment (PTA) results for five imipenem dosing regimens for different MIC targets (0.5, 1, 2, 4, 8 mg/L) for a PD target of 40% fT > 1 × MIC. Relebactam achieved >90% for (ƒAUC0–24 h/MIC) ≥ 7.5 when 1 × MIC is 2 mg/L (4 × MIC = 8 mg/L). Both imipenem and relebactam achieved 100% PTA for 1 × MIC for their PD targets. However, imipenem needed to be dosed at 600 mg every 6 h for the more stringent PD target (4 × MIC).

## 3. Discussion

This is the first study to determine CRRT membrane adsorption and CL_TM_ of imipenem/relebactam. The imipenem CL_TM_ data from this ex vivo study are consistent with several published imipenem pharmacokinetics in vivo studies [8,9,16,17,18].

Our CRRT clearance results with imipenem were consistent with previous reports. Tegeder et al. used CVVH with AN69 hemodiafilter in 12 critically ill patients, and reported a CL_TM_ of 22.9 mL/min (SC of 1.2 with Quf of 18–20 mL/kg/h) [7]. Hashimoto et al. used continuous venovenous hemodialysis with a polyacrylonitrile hemodiafilter and reported a CL_TM_ of 17 mL/min (mean Qd of 20 mL/min) [8]. The most recent study by Boucher et al. determined the pharmacokinetics of imipenem in burn intensive care unit patients undergoing high-dose (mean Quf 53 mL/kg/hour) CVVH [17]. In this in vivo study, the mean SC of imipenem was 1.01, and the CL_TM_ was 54.5 mL/min with the polyethersulfone hemodiafilter.

The package insert [19] for this combination product does not contain any dosing recommendations for critically ill patients receiving CRRT, however it does state that the dose should be reduced to 0.5 g (200 mg of imipenem, 200 mg of cilastatin and 100 mg of relebactam) in patients who receive hemodialysis. This dose for patients receiving hemodialysis is very different from our MCS-derived dose of 1.5 g (600 mg imipenem, 600 mg cilastatin and 300 mg relebactam) for CRRT patients, especially if a very stringent pharmacodynamic target (≥40% fT > 4 × MIC) is to be attained. This dose is higher than what is recommended in the package insert for patients who have a creatinine clearance of >90 mL/min [1.25 g (imipenem 500 mg/cilastatin 500 mg/relebactam 250 mg)]. However, imipenem dose recommendations for critically ill patients with acute kidney injury receiving CRRT have always been consistently higher than what has been recommended for end-stage kidney disease patients. Dose recommendations for imipenem have been either 500 mg every 6 h [6,20] or a loading dose of 1 g and 500 mg every 8 h [21]. The reason for higher doses in acute kidney disease than in end-stage kidney disease appears to be a substantially higher non-renal clearance in patients with acute kidney injury [6,7].

Limitations of this study include the use of bovine blood as the study matrix, instead of human blood. Protein binding could differ as bovine albumin differs from human. The concentration of bovine albumin (~3 g/dL) is not much different from what is often seen in critically ill patients receiving CRRT. Comparisons of previous ex vivo studies, using the same methods as in vivo CRRT trials of the same drug, have shown good agreement in clearance estimates [22,23]. Yet, this study could not perform all possible flow rates, filters, and combinations thereof and, therefore, the findings and conclusions should be limited to the dialysate and ultrafiltration rates conducted in this trial. For MCS, toxicity was not assessed when developing the dosage recommendation. Moreover, the simulation assumed that CRRT ran uninterrupted, and this study only performed commonly utilized PD targets. 

## 4. Materials and Methods

This study assessed drug adsorption and CL_TM_ using CH and CHD configurations. This validated ex vivo CH and CHD models [24,25] and utilized pH regulated, citrate-anti-coagulated bovine blood (Animal Technologies, Tyler, TX., USA). Two hemodiafilters were tested: HF1400 (Polyarylethersulfone, Baxter Healthcare, Deerfield, IL, USA; surface area 1.4 m^2^) and the Multiflow-150 (AN69, Baxter Healthcare, Deerfield, IL, USA; surface area 1.5 m^2^). The Braun-Diapact™ CRRT system (Braun, Bethlehem, PA, USA) tubing set was used. New bovine blood, new hemodiafilters, and new CRRT tubing sets were used in each experiment. The blood was continuously stirred and heated to 37 °C in a water bath during all experiments. Imipenem was reconstituted with a pH controlled stabilizing buffer, formulated with 1:1 of 1.0 M MES (10.88 g 2-(*N*-morpholino) ethanesulfonic acid (MES) and 9.62 g MES sodium salt) and 50% ethylene glycol. Reconstituted imipenem and relebactam (Merck & Co., Kenilworth, NJ, USA) were added to the blood to achieve the final concentration of 20 mg/L (imipenem) and 15 mg/L (relebactam) which approximate plasma peak concentration after dosing. The stabilizing buffer needed to be added in these experiments, since imipenem without cilastatin is unstable, and would degrade. Urea (Lot No. 30K0221; Sigma, St. Louis, MO, USA) was used as a control and added to the blood to produce a blood urea nitrogen (BUN) concentration of ~75 mg/dL, to approximate what might be seen in a critically ill patient with acute kidney injury.

### 4.1. Degradation

Degradation experiments (*n* = 6) were performed to ensure that the imipenem/relebactam was stable when administered in the 37 °C blood for the 1 h experiments without the CRRT apparatus. One liter of bovine blood was prepared identically, as stated above. Urea was added as a control, because it is known to be stable at 37 °C and does not adsorb to hemodiafilters and CRRT circuit [14]. The purpose of this experiment is to account for degradation when determining adsorption clearance and CL_TM_. Blood samples were collected from the flask at 0 (baseline), 5, 10, 20, 30, and 60 min to detect degradation.

### 4.2. Adsorption

The adsorption study (*n* = 6) was performed to determine if imipenem/relebactam would adsorb to the hemodiafilter and/or CRRT circuit. A commonly prescribed blood flow rate (Qb) of 200 mL/min, and an Quf of 33 mL/min were used to observe the decrease in concentration of the drugs in the blood over 1 h. This allowed imipenem/relebactam to have maximal contact with the hemodiafilter circuit and membrane. Since the CRRT machine was primed with normal saline before the operation, the solute dilution caused by the residual priming solution was accounted for. Blood samples were collected from the pre-filter port at 0 (baseline), 5, 10, 20, 30, and 60 min to assess adsorption over time.

### 4.3. Continuous Hemofiltration

The procedures for the CH study (*n* = 6) were similar to those described in our previous ex vivo studies [22,25,26,27,28]. In a closed-loop system, the formed ultrafiltrate was returned to the blood as a post-filter replacement fluid. The CL_TM_ of urea and imipenem/relebactam were evaluated with a commonly used Quf of 17 mL/min, 33 mL/min, and 50 mL/min with two hemodiafilters. All samples were collected after 4 min (Quf 50 mL/min), 6 min (Quf 33 mL/min), and 12 min (Quf 17 mL/min) in randomized order. Pre- and post-hemodiafilter blood samples and ultrafiltrate samples were always collected concurrently.

### 4.4. Continuous Hemodialysis

The procedures for the CHD study (*n* = 6) were similar to those described in our previous ex vivo studies [24,25,27]. The Qb of 200 mL/min with four different dialysate flow rates (Qd) was used: 17, 33, 50, and 100 mL/min. Dialysate was prepared as directed by the manufacturer. Sodium bicarbonate powder (Naturalyte^®^ 4000, Fresenius Medical Care, Waltham, MA, USA) was mixed with distilled water in a 45:1 ratio. After adding imipenem/relebactam, urea and stabilizing buffer to 2 L of blood, the blood was recirculated through the circuit for 5 min, for uniform coating of the CRRT circuit. A single-pass mode of the CHD procedure with four different rates run in random order was performed for each hemodiafilter. Blood samples were collected from the pre-filter and post-filter ports, and spent dialysate samples were taken from the ultrafiltrate port. All samples were collected after 3 min (Qd 100 mL/min), 4 min (Qd 50 mL/min), 5 min (Qd 33.3 mL/min), and 9 min (Qd 16.6 mL/min), in randomized order. Saturation coefficient (SA) and CHD CL_TM_ were calculated. All equations that are used in this study are available in previous studies [22,24,25,26].

### 4.5. Sample Analysis

All blood samples were centrifuged at 3000 rpm for 10 min. The plasma and ultrafiltrate samples were transferred to cryovials in duplicate. Upon collection, the samples were diluted 1:1, with equal parts of ethylene glycol and MES buffer, pH 6.0. These samples were stored at −80 °C until analysis. BUN concentrations were analyzed with Advia 1800 (Siemens Healthcare Diagnostic Inc., Tarrytown, NY, USA) with a lower limit of quantification of 5 mg/dL. Liquid chromatography-mass spectroscopy (LC-MS/MS) was used to determine the concentrations of relebactam and imipenem. Samples, standards, and controls were processed with internal standards and matrix-matched. The typical standard curve range was from 0.005 to 100 µg/mL. Following protein precipitation, the samples were centrifuged, and the supernatant was analyzed using Hydrophilic Interaction Chromatography (HILIC), and detected via tandem mass spectrometry. Liquid chromatography was performed on a Thermo Fisher Scientific Transcend™ II multiplexed ultra-high-performance liquid chromatography system, using a Waters Atlantis HILIC column (3 µm, 2.1 mm × 50 mm). Elution was performed using gradient elution with 0.1% formic acid in water (mobile phase A), and 1% of 10 mM ammonium acetate pH 4.5 in acetonitrile (mobile phase B). The HPLC system was coupled to Applied Biosystems-Sciex 4500 triple quadrupole tandem mass spectrometers (Foster City, CA, USA) equipped with an electrospray source operating in positive ion mode, using multiple reaction monitoring

### 4.6. Monte Carlo Dosing Simulations

All doses were given every 6 h and were available as imipenem/cilastatin/relebactam. Dosing regimens of 0.5 grams (imipenem 200 mg/cilastatin 200 mg/relebactam 100 mg), 0.75 grams (300 mg/300 mg/150 mg), 1 gram (400 mg/400 mg/200 mg), 1.25 grams (500 mg/500 mg/250 mg), and 1.5 grams (600 mg/600 mg/300 mg) were simulated in the MCS for the first 48 h [29]. Cilastatin was not simulated, due to its lack of PK data and clinical impact. Drug concentration-time profiles were generated in a log-Gaussian distribution with preset limits, using the mean and SD of the pharmacokinetic parameters [e.g., weight (range of 40 kg to no maximum), Vd, free fraction, CLNR, SC] by the MCS program (Crystal Ball, Oracle©, Irvine, CA, USA) in 5000 virtual subjects for each dosing regimen (Table 2). The continuous venovenous hemofiltration (CVVH) regimen was modeled with an effluent rate of 25 mL/kg/h which is KDIGO recommended CRRT rate [30]. The values for CLNR and Vd were obtained from the published clinical studies (imipenem and relebactam), or from internal manufacturer data (relebactam). Lastly, the correlations (r^2^) between body weight and Vd or CLNR were derived and incorporated into the model for imipenem, based on available PK data (Table 3).

### 4.7. Pharmacodynamic Targets

The PD targets were: ≥40% fT > MIC (≥40% fT > 4 × MIC) for imipenem, and the free drug area under the concentration–time curve from 0 to 24 h to MIC (ƒAUC0–24 h/MIC) and ≥7.5 for relebactam, respectively, for the first 48 h of antibiotic therapy [13,14,31]. The clinical breakpoint of *P. aeruginosa* for imipenem and relebactam was 2 mg/L [14]. Thus, we evaluated the attainment of PD targets of ≥40% fT > MIC of 2 mg/L (4 × MIC = 8 mg/L for imipenem), and (ƒAUC0–24 h/MIC) ≥7.5 of 2 mg/L (4 × MIC = 8 mg/L for relebactam) for the first 48 h of antibiotic therapy, to determine the optimal dosing regimen [14]. A second, more stringent PD target (4 × MIC and 100%fT < MIC) was analyzed, due to data supporting a higher MIC target yielding benefits in critically ill patients [32].

### 4.8. Optimal Dosing Regimen

A probability of target attainment (PTA) of 90% has been used as a standard threshold to determine the optimal drug dosing regimen; it was used as the goal in this study [33,34]. A PTA of 90% means the MCS predicts that the dosing regimen will ensure that 90% of the virtual patients attained the predetermined pharmacodynamic target.

### 4.9. Data Analysis

A power analysis calculation indicated that six experiments were required to detect a 25% difference in the extent of imipenem/relebactam adsorption. Similarly, six CH and six CHD experiments with each hemodiafilter were required to detect a 25% difference in imipenem/relebactam CL_TM_ between hemodiafilters [http://powerandsamplesize.com/Calculators/Compare-2-Means/2-Sample-Equality, accessed on 1 August 2017]. Assumptions used in these calculations included: 90% power and 10% standard deviation with a significance level of *p* < 0.05. A two-tailed, unpaired t-test was used to compare differences between the two hemodiafilters, and analysis of variance was used to compare the different Quf and Qd within each hemodiafilter type.

## 5. Conclusions

Imipenem/relebactam is unlikely to be adsorbed by the hemodiafilter, but will be readily removed by CRRT. Relebactam will reach the PD target at all times when imipenem reaches its PD target with CRRT effluent rates of 25 kg/mL/h. Our MCS suggests that 200 mg of imipenem every 6 h reached the PD target of fT40% > 1 × MIC in 90% of virtual patients receiving the dose. However, 600 mg every 6 h was required to attain a stringent PD target (4 × MIC). Relebactam was able to achieve the PTA for a stringent PD target at any recommended dose for CRRT. This study shows that imipenem and relebactam will likely require dose adjustments for critically ill patients receiving CRRT.

## Figures and Tables

**Table 1:** Mean sieving coefficients and saturation coefficients of imipenem and relebactam.

Effluent Rate (mL/min)	HF1400 (*n* = 6, Mean ± SD)		M150 (*n* = 6, Mean ± SD)	
	Imipenem	Relebactam	Imipenem	Relebactam
Sieving coefficients				
16.7	1.06 ± 0.2	0.95 ± 0.08 *	1.19 ± 0.2	1.07 ± 0.06 *
33.3	1.1 ± 0.2	1.00 ± 0.04 *	1.1 ± 0.2	1.08 ± 0.1 *
50	1.01 ± 0.2	0.94 ± 0.08	1.3 ± 0.2	1.12 ± 0.06
Saturation coefficients				
16.7	1.2 ± 0.3	1.2 ± 0.06 *	0.9 ± 0.4	0.9 ± 0.2 *
33.3	1.7 ± 1.3	1 ± 0.2	0.99 ± 0.5	0.9 ± 0.5
50	1.2 ± 0.3	1.1 ± 0.1	1.23 ± 0.1	1.1 ± 0.1
100	1.1 ± 0.2	1.02 ± 0.1 *	1.17 ± 0.2	0.9 ± 0.05 *

* The *t*-test between hemodiafilters (HF1400 vs. M150) was *p* < 0.05 at this flow rate.

**Table 2:** Demographic and Pharmacokinetic Parameters Used in Monte Carlo Simulations.

Pharmacokinetic Parameters	Imipenem [12]	Relebactam [13,14]
Vd (L/kg)	0.34 ± 0.1 (0.21−0.63)	0.19 ± 0.11 (0.08−0.53)
Free Fraction	0.8 ± 0.16 (0−1)	0.78 ± 0.2 (0−1)
NR CL (mL/min)	100.5 ± 28 (53−160)	11.7 ± 3.3 (11.2−12.2)
Sieving coefficient	1 ± 0.2 (0−1)	1 ± 0.04 (0−1)
r^2^ weight & Vd	0.17	N/A
r^2^ weight & NR CL	0.013	N/A
Weight ± SD (kg)	86.6 ± 29.2 (40-∞) [15]	

All values are mean ± standard deviation (minimum–maximum limits). Abbreviations: CL = clearance; N/A = not available; NR = non-renal; r^2^ = correlation; Vd = volume of distribution; Q_eff_ = effluent flow rate; Q_rep_ = replacement fluid flow rate.

**Table 3:** Probability of Target Attainment for Imipenem with Monte Carlo Simulation Approach in Modeled CRRT Patients.

Antimicrobial Dosing	40% fT > 1 × MIC	40% fT > 4 × MIC	100%fT > MIC
Imipenem			
200 mg q6h	100%	0%	0%
300 mg q6h	100%	2%	0%
400 mg q6h	100%	28.6%	33.5%
500 mg q6h	100%	66.1%	60%
600 mg q6h	100%	90.8%	77.2%

This table is for imipenem only, does not include cilastatin and relebactam.

## Data Availability

Data is available and stored at University of Michigan College of Pharmacy.
